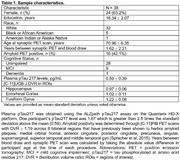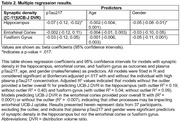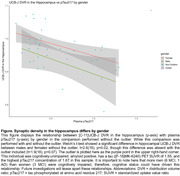# Plasma pTau217, gender, and synaptic density

**DOI:** 10.1002/alz.091468

**Published:** 2025-01-09

**Authors:** Kao Lee Yang, Alexandra H DiFilippo, Yue Ma, Rachael E Wilson, Yer Thor, Mary‐Elizabeth Pasquesi, Todd E Barnhart, Jonathan W Engle, Tobey J. Betthauser, Nicholas J. Ashton, Sterling C. Johnson, Bradley T. Christian, Henrik Zetterberg, Barbara B. Bendlin

**Affiliations:** ^1^ Wisconsin Alzheimer's Disease Research Center, University of Wisconsin School of Medicine and Public Health, Madison, WI USA; ^2^ Department of Medical Physics, University of Wisconsin, Madison, WI USA; ^3^ Wisconsin Alzheimer's Institute, University of Wisconsin School of Medicine and Public Health, Madison, WI USA; ^4^ University of Wisconsin School of Medicine and Public Health, Madison, WI USA; ^5^ Wisconsin Alzheimer’s Institute, University of Wisconsin‐Madison School of Medicine and Public Health, Madison, WI USA; ^6^ Department of Psychiatry and Neurochemistry, Institute of Neuroscience and Physiology, The Sahlgrenska Academy, University of Gothenburg, Mölndal, Gothenburg Sweden; ^7^ Wisconsin Alzheimer’s Institute, University of Wisconsin School of Medicine and Public Health, Madison, WI USA; ^8^ Geriatric Research Education and Clinical Center, William S. Middleton Memorial Veterans Hospital, Madison, WI USA; ^9^ Department of Medical Physics, University of Wisconsin‐Madison, Madison, WI USA; ^10^ Hong Kong Center for Neurodegenerative Diseases, Hong Kong China; ^11^ Department of Neurodegenerative Disease, UCL Institute of Neurology, London UK; ^12^ UK Dementia Research Institute, University College London, London UK

## Abstract

**Background:**

Synaptic loss is a key feature of Alzheimer’s disease (AD) dementia. In the entorhinal cortex (ERC) and hippocampus, phosphorylated tau (pTau) colocalizes with synaptosomes, and its presence may play a role in AD‐related synaptic loss. However, the relationship between pTau and synaptic density is not well understood. Plasma pTau represents secreted tau pathology and among the available epitopes, pTau217 has emerged as an accurate biomarker of AD pathology. Here, we tested the relationship between secreted pTau217 and synaptic density.

**Method:**

Participants were recruited from the Wisconsin Alzheimer’s Disease Research Center and the Wisconsin Registry for Alzheimer’s Prevention (N=38; Table 1). All participants underwent blood sampling for measurement of plasma pTau217 and [C‐11]UCB‐J PET to assess synaptic density in regions of interest (ROIs: hippocampus, ERC, and fusiform gyrus) known to show early AD tau accumulation. Synaptic density was quantified using [C‐11]UCB‐J DVR (LGA, whole cerebellar reference region) and ROIs were identified following FreeSurfer T1w‐MRI parcellation. Plasma pTau217 was determined using the ALZpath pTau217 Simoa assay on the Quanterix HD‐X platform. We utilized multiple regression analysis to examine the extent to which plasma pTau217 and gender predict synaptic density in ROIs controlling for age. All models were fitted in R and considered significant at Bonferroni‐corrected (.05/3) p<.017. During analysis we discovered one outlier; a cognitively‐unimpaired participant with pTau217 concentration >2.5 standard deviations above the mean and high UCB‐J DVR in all ROIs. Results with and without the outlier were considered.

**Result:**

Plasma pTau217 (b=‐.07, p=0.01) and gender (b=‐.05, p=0.008) predicted UCB‐J DVR in the hippocampus, but not other ROIs (Table 2). The effect size of plasma pTau217 and gender, as measured by Cohen’s f^2^, was 0.23 and 0.34, respectively, indicating medium to large effects. Welch’s t‐test showed a significant gender difference in hippocampal UCB‐J DVR (Figure).

**Conclusion:**

Our results indicate that higher levels of plasma pTau217 associated with lower synaptic density in the hippocampus. However, given the individual with high pTau217 and synaptic density, it is possible other processes unaccounted for in this analysis are impacting this relationship. Further examinations could give insight into early processes that confer neuronal injury in the AD pathological cascade.